# An Accumulation Pretreatment-Free POCT Biochip for Visual and Sensitive ABO/Rh Blood Cell Typing

**DOI:** 10.3390/bios15110731

**Published:** 2025-11-02

**Authors:** Pengcheng Wang, Mingdi He, Yan Ma, Yunhuang Yang, Rui Hu

**Affiliations:** 1 State Key Laboratory of Magnetic Resonance Spectroscopy and Imaging, Innovation Academy for Precision Measurement Science and Technology, Chinese Academy of Sciences—Wuhan National Laboratory for Optoelectronics, Huazhong University of Science and Technology, Wuhan 430074, China; 2 Wuhan Blood Center, Wuhan 430030, China; 3 University of Chinese Academy of Sciences, Beijing 100190, China

**Keywords:** point-of-care testing, blood typing, microfluidic chip

## Abstract

Rapid blood type detection in point-of-care testing (POCT) scenarios is crucial for various clinical treatments. In this study, we present a sensitive, cost-effective, and straightforward biosensing approach for visual blood typing that notably simplifies the procedure by eliminating any need for blood sample pretreatment. Our technique achieves this by directly trapping and accumulating red blood cell (RBC) clusters within a photolithography-based microfluidic chip, thereby bypassing complex preprocessing. By employing an antigen–antibody assay involving isoagglutinins A, B, and/or D on the RBC surface and their corresponding antibodies, we effectively determine blood types. When antibodies are present, the corresponding RBCs bind to the antibody-conjugated RBC clusters, which are subsequently trapped within the microfluidic accumulation chip, resulting in the formation of a visible bar. The blood group can then be readily identified by observing this visual bar with the naked eye or under microscopy. Notably, we integrate two continuous mixing units (Z and S) at the entrance of the biochip to improve mixing efficiency and accelerate the antigen–antibody interaction. This method demonstrates high selectivity, accuracy, and stability across various clinical blood samples. Moreover, the sensor operates with minimal sample volume (as low as 10 μL) and delivers results within 5 min. The fabrication cost of the PDMS-based biochip is approximately $0.2 per chip, and the limit of detection (LOD) is determined to be 3 × 10^6^ cells/mL, indicating excellent sensitivity and affordability for practical use. Overall, this biochip provides a fast, low-cost, and reliable solution for emergency blood typing, particularly in resource-limited settings.

## 1. Introduction

Blood transfusion remains a cornerstone of modern clinical practice, particularly for managing massive bleeding, severe anemia, and coagulation disorders. Globally, it is estimated that more than 75 million units of blood are collected and transfused annually to address diverse medical needs, often in critical life-saving situations [1]. Consequently, accurate compatibility testing between donor and recipient blood groups is essential, since mismatched transfusions may trigger agglutination and fatal outcomes [2]. The International Society of Blood Transfusion (ISBT) has established multiple classification systems for blood groups, among which the ABO and RhD systems are of greatest clinical significance. These types are determined by the presence or absence of isoagglutinins A, B, and/or D on red blood cell (RBC) membranes [3]. Currently, micro-column gel testing is the most frequently adopted method for blood typing [4]. Nevertheless, this technique demonstrates limited sensitivity, often failing to detect weak subgroup variants or subtle antigen–antibody interactions [5]. In contrast, advanced technologies—such as flow cytometry, DNA sequencing, and microarray-based genotyping—offer enhanced reliability and sensitivity [6,7]. However, these approaches require sophisticated equipment, trained specialists, and substantial financial resources, making them impractical for bedside or point-of-care use. Importantly, the absence of a robust, rapid pre-transfusion assay continues to contribute to reports of ABO-incompatible transfusions worldwide each year [8]. Therefore, there is an urgent need to develop reliable, highly sensitive, and cost-effective point-of-care testing (POCT) platforms for transfusion safety.

Point-of-care testing (POCT) is frequently required in transfusion practice [9], enabling blood group determination at the bedside or in non-laboratory environments such as fieldwork and emergency care. Hemagglutination remains one of the earliest and most fundamental POCT methods, in which specific antibodies trigger visible agglutination of red blood cells (RBCs) in a liquid medium [10]. In recent years, biosensor-based analytical platforms have been explored as alternatives for blood typing. For example, Hertaeg and colleagues developed a paper-based assay by impregnating filter paper with RBC-specific antibodies, resulting in the appearance of a plasma separation band when blood agglutination occurred [11]. Similarly, Whitesides and co-workers introduced a microfluidic paper-based device fabricated using photolithography, where hydrophobic and hydrophilic domains were patterned to guide reactions for blood group identification [12]. Although these paper-based and microfluidic systems are promising for POCT, their applications remain limited, as they generally detect only ABO and/or RhD antigens. In fact, identifying the subgroup variant in blood samples with the POCT device had been a largely under explored domain. It is well known that blood group A has two kinds of subgroup variants, A1 and A2. Although A1 and A2 subgroups are managed similarly in transfusion medicine, they differ markedly in the density of A antigens on the red blood cell surface. Specifically, A1 erythrocytes typically carry approximately 0.8–1.2 × 10^6^ A antigens per cell, whereas A2 cells express only about 2.4–2.9 × 10^5^ antigens [13]. Similarly, A reduction in the number of red blood cells in blood samples can also lead to a decrease in the number of antibodies. Therefore, the reducing numbers of RBCs in the blood samples are the major obstacle to blood type identification.

Lab-on-a-chip technology has emerged as one of the most promising approaches for clinical sample detection in point-of-care testing (POCT), and microfluidics-based biosensors are among the most widely employed methods for implementing such assays. Microfluidic devices have been applied in various fields, such as drug screening, protein renaturation, DNA sequencing, cell analysis, chemical synthesis, and others [14,15,16,17]. Among various types of micro-devices, the PDMS-based device is the most popular one utilized in biochemical analysis due to its good biocompatibility. It should be noted that a PDMS-based microfluidic biochip can be fabricated by photolithography, which means that its structure could be designed to solve specific problems according to experimental requirements [18]. Moreover, rapid mixing of reactants can be achieved by designing micro-mixing structures in microfluidic chips, thus improving biosensing sensitivity [19].

In this study, we present a simple, sensitive, and versatile microfluidic biochip for red blood cell accumulation, enabling visual blood typing that can be assessed both microscopically and by the naked eye. When RBCs and their corresponding antibodies mixed in the microfluidic chip, the RBCs could bind to antibodies, forming a large cluster flowing in the microfluidic chip. Of note, to demonstrate the ability of the biochip to detect the type of blood, the diluted blood samples testing was investigated. This work presents an economical, stable, and robust strategy for low-cost visual blood typing, highlighting its strong potential for clinical application.

## 2. Materials and Methods

### 2.1. Materials

Fluorescein and sulforhodamine B (fluorescent dyes), analytical-grade inorganic salts, and buffer reagents (HCl, Tris, and EGTA) were obtained from Sinopharm Chemical Reagent (Shanghai, China). Fluorescein (MW 500,000) and tetramethylrhodamine-labeled dextrans (MW 70,000) were obtained from Molecular Probes (Eugene, OR, USA). All experimental solutions were prepared using dd-water (Millipore, Bedford, MA, USA). Antibodies, including anti-A, anti-B, and anti-D (Rh), were provided by the Wuhan Blood Center (Lots: 57,014, 57,033, 57,011, Shanghai, China). Nine EDTA-anticoagulated RBC samples were supplied by the Wuhan Blood Center, and their blood groups were confirmed by micro-column gel assays. All procedures were approved by the Wuhan Blood Center Ethics Committee (No. KY2025-101).

### 2.2. Device Fabrication

Microfluidic biochips designed for red blood cell (RBC) accumulation were produced through conventional soft photolithography. To generate the master mold, a negative photoresist (SU-8 3025, MicroChem, MA, USA) was applied onto a clean silicon wafer by spin coating at 800 rpm for 40 s followed by 1500 rpm for 60 s. The coated wafer underwent a two-step prebake at 65 °C for 15 min and 95 °C for 35 min. The spin-coated wafer was subjected to ultraviolet (UV) exposure for 10 s at 5 mJ/cm^2^ and then post-baked at 95 °C for 10 min. Development of the photoresist pattern was achieved in propylene glycol methyl ether acetate (PGMEA) for approximately 3 min, followed by rinsing with isopropanol. To enhance structural stability, the wafer underwent a hard bake at 135 °C for 60 min, producing the final SU-8 mold. For polydimethylsiloxane (PDMS) fabrication, the base polymer and curing agent (10:1, *w*/*w*) were mixed, degassed under vacuum, and poured over the SU-8 mold. The mixture was cured at 65 °C for 2 h to form a PDMS mold incorporating the designed microchannel structures. After curing, the PDMS layer was cut, carefully peeled from the SU-8 master, and inlet/outlet holes were introduced using a biopsy punch. The PDMS layer and a glass substrate were then treated with oxygen plasma and permanently bonded, resulting in sealed microfluidic devices suitable for subsequent experiments.

### 2.3. Characterization of Microfluidic Red Blood Cell Accumulation Biochips

Microfluidic blood red cell accumulation biochips were characterized with a scanning electron microscope (SEM, FEG-SU8030, Tokyo, Japan) for surface morphology information. In the present study, the mixing unit, the reaction chamber, and the biosensing channel, which were located in the biochip, were selected for the surface characterization.

### 2.4. Visual RBC Blood Typing Using the Microfluidics Biochip

Blood typing in the microfluidic biochip was achieved through an antibody–antigen reaction assay. In brief, 10 μL of blood sample and 10 μL of antibody solution (anti-A, anti-B, or anti-D) were introduced into the designated inlets (A/B). A needle connected to the outlet was coupled to a vacuum pump to generate uniform and stable negative pressure, thereby driving the sample and antibody solutions through the inlet, mixing unit, and reaction chamber into the biosensing channel. The results could be directly observed within the biosensing channel, and the entire assay was completed in less than 5 min.

Moreover, when measuring the capability of the biochip, the whole blood was diluted with serial concentrations from 1.2 × 10^7^ to 7.5 × 10^5^ cells/mL, and the diluted sample was applied on the biochip for blood typing.

### 2.5. Optical Imaging System and Image Analysis

The biosensing channels of the biochip were imaged and characterized using an EVOS FL Auto 2 imaging system (Thermo Fisher Scientific, Waltham, MA, USA). This platform enables the acquisition of bright-field and fluorescence images as well as real-time videos, and supports large-area imaging with automated image stitching. Fluorescence signals from fluorescein-labeled dextran and tetramethylrhodamine-labeled dextran were recorded. Quantitative analysis of fluorescence intensity was performed using ImageJ software 1.45s.

### 2.6. Statistical Analysis

Scanning electron microscopy (SEM) images were analyzed to obtain surface information using ImageJ software 1.45s (NIH, Bethesda, MD, USA). Statistical analyses, including linear regression, curve smoothing, differentiation, and integration, were carried out with Origin software 2022b (9.95) to process experimental data and generate graphs.

## 3. Results and Discussion

### 3.1. Working Principle of the Microfluidic Red Blood Cell Accumulation Biochip

**Figure 1:**
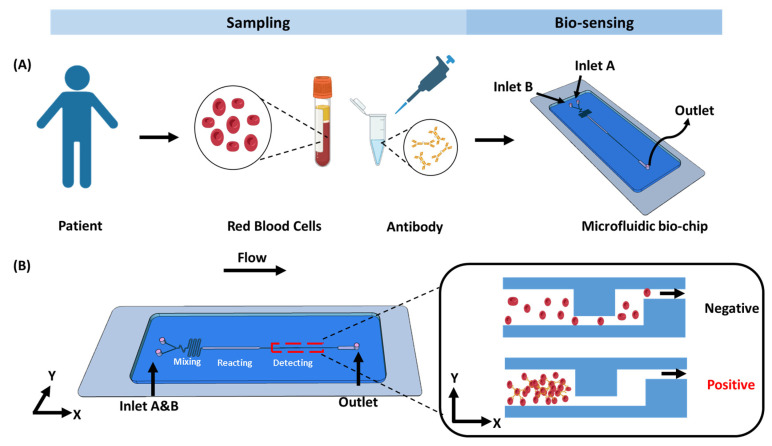
Working principle of the biochip. (**A**) Overview of the steps involved in the detection process in blood typing. (**B**) On-chip assay, schematic of the microfluidic biochip designed for RBC trapping and accumulation.

**Figure 2:**
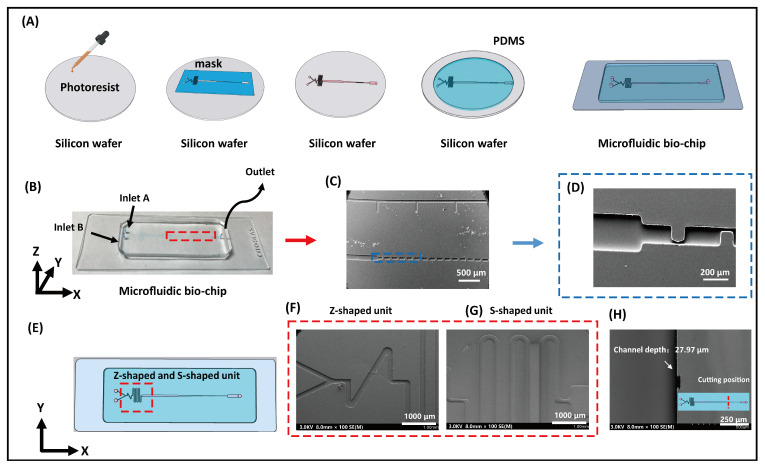
(**A**) The schematic of standard soft photolithography fabrication biochip. (**B**) Photograph of the microfluidic accumulator biochip. (**C**,**D**) SEM images of biosensing channel of biochip, corresponding to the area marked with dashed lines in the (**B**) schematic. (**E**) Schematic illustration of the microfluidic chip. (**F**,**G**) SEM images of Z- and S-shaped unit. (**H**) SEM image of depth of biochip.

To visually identify RBC types, we designed a microfluidic accumulator biochip (Figure 1 and Figure 2) consisting of three main functional regions: a mixing channel, a reaction chamber, and a biosensing channel. Specially, the mixing channel is composed of two geometrically distinct subunits, which are hereafter referred to as the Z-shaped unit and S-shaped unit. As shown in Figure 2E–G, two continuous mixing units (Z-shaped and S-shaped) are positioned as the first part of the biochip. For the mixing channel, Z- and S-shaped units are designed to divide the laminar flow from the inlets and each of the sharp corner parts of the Z and S units bends back to block a portion of the laminar flow and induce fluid turbulence, which can greatly change the flow direction in all directions and efficiently mix the fluids. Additionally, the reaction chamber with larger channel width and length could provide sufficient space for RBCs to bind with specific antibodies forming a large cluster. The core functional element of the RBC accumulation biochip is the biosensing channel, which is composed of two interconnected microchannel layers: a wider channel (50 μm) and a narrower channel (10 μm). This structural design enables the effective trapping and packing of red blood cell (RBC) clusters with diameters greater than 10 μm, as they become retained at the junction formed by the width difference. In contrast, individual RBCs of smaller sizes can freely pass through the microchannels without obstruction. Importantly, to ensure optimal biosensing performance, the depth of the microfluidic channel was precisely maintained within 10–30 µm, corresponding to approximately two to three times the diameter of a red blood cell. This dimensional constraint enables the passage of individual RBCs while effectively restricting larger cell aggregates. Surface analysis by SEM (Figure 2H) confirmed that the fabricated microfluidic chip exhibited a uniform channel depth of 27.97 µm, and the dimensional deviation was within the acceptable fabrication tolerance. This design offers an efficient trapping platform with strong resistance to clogging. As a result, RBC clusters can be feasibly retained and packed within the biosensing channel (Video S1), producing a distinct red visual bar. The blood type can then be directly identified by observing this bar with the naked eye or under a microscope. It should be noticed that the detailed structures of the photomask are provided in Figure S1.

### 3.2. Characterization of the Microfluidic Red Blood Cell Accumulation Biochip

The design, fabrication, and experimental procedures of the RBC-typing microfluidic accumulator biochip are illustrated in Figure 2. Notably, the PDMS-based biochip was fabricated using a standard soft lithography technique, which enables the precise formation of microchannels at the microscale. The design, fabrication, and experimental procedures of the RBC-typing microfluidic accumulator biochip are illustrated in Figure 2.

To verify that the microfluidic channels were fabricated as designed, scanning electron microscopy (SEM) was employed to observe the channel morphology. As shown in Figure 2, SEM imaging confirms the structural integrity and configuration of the biochip. Two solutions can be independently introduced through the A and B inlets, converge at a Y-shaped junction, and flow through a serpentine mixing region composed of alternating Z- and S-shaped units. After thorough mixing, the solutions reach the entrance of the reaction chamber. Importantly, Figure 2 clearly demonstrates that the biosensing channels closely match the intended design, benefiting from the high-resolution capabilities of soft lithography. Given that the diameter of red blood cells (RBCs) ranges from approximately 7 to 10 μm, the biosensing region was engineered with two channel widths—50 μm and 10 μm—to allow single RBCs to pass through while trapping aggregated RBC clusters for visual detection.

### 3.3. Evaluation of Mixing Channel of the Microfluidic Red Blood Cell Accumulation Biochip

**Figure 3:**
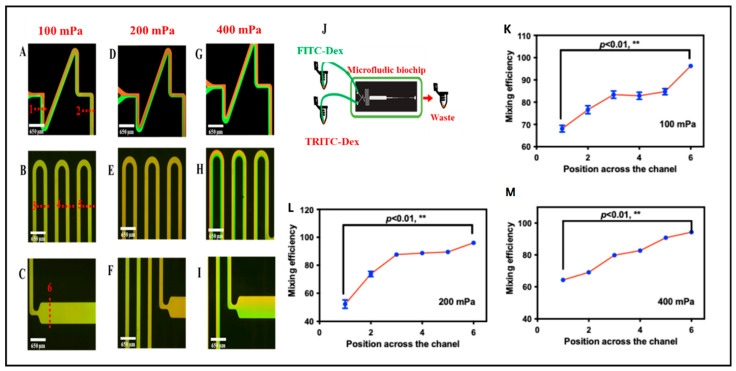
(**A**–**I**) Representative mixing of fluorescein–dextran and tetramethylrhodamine–dextran in the microchannel at various flow rates. The overlaid dotted lines indicate predefined sampling positions along the channel; these positions correspond to the x-axis locations used in (**K**–**M**). (**J**) Schematic diagram of the microfluidic biochip. (**K**–**M**) Mixing efficiency of the biochip at different flow rates and different locations (** *p* < 0.01, *t* test and nonparametric tests).

To evaluate the performance of the mixing channel, the mixing efficiency of fluorescein was quantified under different outlet negative pressures ranging from 100 mPa to 400 mPa. The negative pressure was supplied by a pneumatic pump, which allowed precise adjustment of the pressure level (Figure S2). The fluorescence distribution along the y-axis of the channel is presented in Figure 3. At an outlet negative pressure of 400 mPa, a distinct fluid interface was observed at the entrance of the reaction chamber, with only limited mixing occurring between the two solutions. As the pressure was reduced to 200 mPa and 100 mPa, turbulent flow developed, promoting enhanced mixing. Remarkably, at these lower pressures, complete mixing was achieved within the mixing channel. Figure 3 illustrates the fluorescence distributions under different outlet pressures at the entrance of the reaction chamber.

To quantitatively evaluate mixing performance, the mixing efficiency (*C_m_*) was determined using the following equation:

(1) $$C_{m}=1-\frac{\sqrt{{\Sigma (x_{i}-\bar{x})}^{2}/N}}{\bar{x}}$$

where *x_i_* is the fluorescence intensity of each pixel in the cross section, N is the number of total pixels, and $\bar{x}$ is the average fluorescence of all the pixels. Larger *C_m_* indicated better mixing. Figure 3 demonstrates that when the negative pressure decreased to 200 and 100 mPa, *C_m_* at the entrance of the reaction chamber reached 93% and 95%, respectively, suggesting that uniform fluorescence was achieved. To confirm the mixing efficiency, the blood sample and the corresponding antibodies were selected. At the negative pressure of 100 and 200 mPa, the optical microscope images show that the RBCs were homogeneously distributed in the mixing channel, which indicated that blood samples and antibody solutions could be mixing well under 100 and 200 mPa in the biochip. However, when aggregated RBCs pass through the sensing channel, clumps with faster flow rates (with 200 mPa negative pressure) were washed away when they hit the walls of the sensing channel. Fortunately, clusters with lower flow rates (with 100 mPa negative pressure) could be successfully trapped and packed in the biosensing channel. Therefore, in the present study, we selected 100 mPa to drive the fluid in the biochip to obtain both good mixing efficiency and biosensing ability.

### 3.4. ABO and RhD Blood Group Typing on the Microfluidic Red Blood Cell Accumulation Biochip

**Figure 4:**
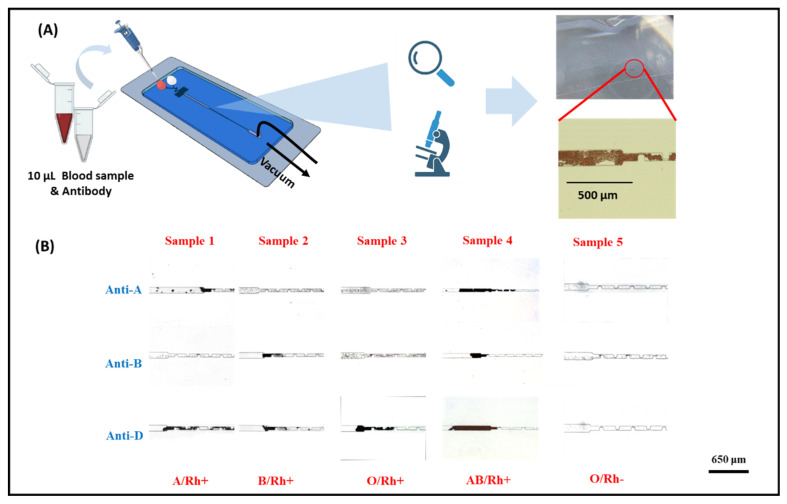
Integration of antigen–antibody-based assay on microfluidic accumulator biochip for blood typing. (**A**) Schematic of the steps involved in the detection process in blood typing. (**B**) Testing results of clinical blood samples for blood typing.

**Figure 5:**
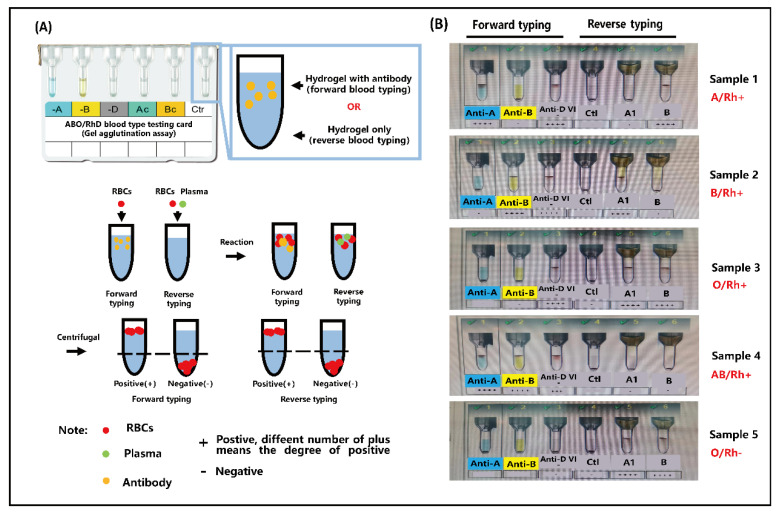
(**A**) Schematic of the testing protocols of micro-column gel in blood typing. (**B**) The testing results of blood samples for blood typing by micro-column gel.

ABO and RhD blood grouping was qualitatively evaluated by visual inspection, either with the naked eye or under a microscope. Blood groups A (Rh^+^), B (Rh^+^), AB (Rh^+^), O (Rh^+^), and O (Rh^−^) were tested using 10 μL of whole blood and 10 μL of the corresponding antibodies (anti-A, anti-B, and anti-D). In the experimental setup, small volumes (10 μL) of blood samples and antibodies were separately introduced into the A or B inlets of the biosensor. Simultaneously, negative pressure was applied at the outlet of the microfluidic biochip using a pneumatic pump to drive the sample through the chip. The entire assay was completed within 5 min. It is worth noting that a visible red band, formed by trapped RBC clusters in the sensing channel, was observed either with the naked eye or under a microscope, indicating a positive reaction. These RBC clusters result from the specific recognition between the surface antigens on RBCs and their corresponding antibodies. To further evaluate the applicability of the developed biosensor, nine clinical blood samples were examined for ABO/Rh blood typing, as illustrated in Figure 4 and Figure S4. As shown in Figure 4, only RBC-A and RBC-AB samples reacted with anti-A, leading to RBC clustering and entrapment within the chip. This is attributed to the presence of antigen A on the membranes of RBC-A and RBC-AB. Similarly, Figure 4 illustrates the specific interaction between anti-B and RBC-B or RBC-AB, which also results in visible clustering. In contrast, RBC-O, which lacks both antigens A and B, does not interact with either anti-A or anti-B and thus does not form clusters. Furthermore, when anti-D is present in the system, the Rh blood type can be determined: a red visual bar indicates an Rh-positive (Rh^+^) blood type, while no visible bar indicates Rh-negative (Rh^−^). All results were easily distinguished by the naked eye or under microscopy, as demonstrated in Figure 4. Moreover, as shown in Video S1, blood cell aggregation occurs within the microfluidic channel, and the aggregated cells are subsequently captured by the sensing region of the biochip, providing a direct visualization of the detection process. Importantly, the blood typing results obtained from the microfluidic biochip were fully consistent with those obtained using the micro-column gel test (Figure 5). The blood typing results obtained using the biochip and those obtained using the micro-column gel method are summarized in Table S1. In summary, the red visual bar formed by RBC trapping and accumulation provides a reliable, low-volume, and rapid method for blood group typing.

### 3.5. Determination of the Red Blood Cell Accumulation Biochip Detection Limit

**Figure 6:**
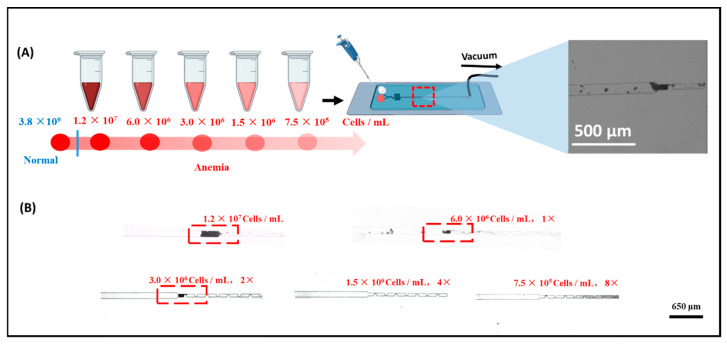
(**A**) Schematic of the steps involved in the dilution process in blood typing ability test. (**B**) Testing results of diluted blood samples for blood typing.

To further evaluate the performance of the biochip, its detection limit for red blood cells (RBCs) was investigated (Figure 6). RBC-A samples were serially diluted to obtain solutions with RBC concentrations ranging from 1.2 × 10^7^ to 7.5 × 10^5^ cells/mL. The cell concentration was measured using an automatic cell counter (Figure S3), with three parallel experiments performed. Each concentration was tested for 5 min, and changes in the biosensing channel were monitored throughout the interaction process. As shown in Figure 6, a visible red band formed when the concentration of RBCs exceeded 3 × 10^6^ cells/mL. In contrast, no measurable changes were observed when the concentration decreased to 1.5 × 10^6^ cells/mL. Based on these results, the limit of detection (LOD) of the RBC accumulation biochip is estimated to be approximately 3 × 10^6^ cells/mL. It should be noted that the red blood cell concentration in normal human blood greatly exceeds 3 × 10^6^ cells/mL, which is the detection limit of the present biochip, demonstrating its applicability for clinical blood typing. Therefore, this biochip is suitable for blood typing in patients with anemia or erythrocytopenia. Moreover, as shown in Table S2, the present study reports a microfluidic biochip for rapid and cost-effective ABO/Rh blood type detection in point-of-care testing (POCT). The proposed biosensor accomplishes blood typing within 5 min using less than 10 μL of whole blood, demonstrating excellent suitability for POCT applications. In comparison, a paper-based assay developed by Julauk et al. [20] required approximately 10 min for detection and exhibited limited accuracy, achieving only 85% accuracy for type B blood typing. Similarly, Wang et al. [21] reported an LSPR-based blood typing biosensor that required up to 1 mL of blood sample for analysis. In contrast, our biochip requires only 10 μL of blood while maintaining a higher accuracy rate, faster detection speed, and a lower limit of detection (LOD) than the LSPR-based method. Overall, the developed chip exhibits superior performance in terms of sensitivity, efficiency, and sample economy, making it a promising platform for clinical POCT applications.

## 4. Conclusions

Rapid detection of blood type in point-of-care testing (POCT) scenarios is of considerable importance for a wide range of clinical applications. In the present study, we successfully demonstrate a cost-effective, sensitive, and straightforward approach for the visual identification of RBC blood groups, which can be directly interpreted either by the naked eye or under a microscope. Meanwhile, the limit of detection (LOD) of the RBC accumulation biochip is estimated to be approximately 3 × 10^6^ cells/mL and it could realize in 5 min. Moreover, when the present biochip was applied for clinical sample application, the testing results were 100% consistent with the slide agglutination assay, which indicated that our present detection methods are highly reliable. Here, we believe that our present biochip could provide a sensitive, visual, and economic clinical blood typing analysis.

## Data Availability

The raw data supporting the conclusions of this article will be made available by the authors on request.

## Supplementary Materials

The following supporting information can be downloaded at https://www.mdpi.com/article/10.3390/bios15110731/s1, Figure S1: (A) Image of biochip lithography mask. (B) Biochip design dimensioning diagram; Figure S2: (A) Image of testing system of blood type biochip. (B) Tubing connection between biochip and pneumatic pump. (C) Display panel of pneumatic pump showing the negative pressure. (D) Image of biochip under working conditions; Figure S3: (A) Image of automatic cell counter and cell counting chamber. (B) Testing report of red blood cells (RBCs) obtained using the Cellometer K2 Cytometer. (C) Images showing different RBC concentrations; Figure S4: Testing results of clinical blood sample for blood typing; Figure S5: Image of biochip filled with colored dye; Figure S6: Testing results of clinical blood sample for blood typing; Table S1: Comparison of blood typing results obtained using the developed microfluidic biochip and the classical gel column method; Table S2: Comparison with existing POCT blood typing technologies; Video S1: Blood type chip captures red blood cell agglutination.
