# Supplementary material for: An Accumulation Pretreatment-Free POCT Biochip for Visual and Sensitive ABO/Rh Blood Cell Typing
Source: Biosensors (Basel). 2025 Nov 2;15(11):731. doi: 10.3390/bios15110731 (PMC12650352; doi:10.3390/bios15110731)
Supplement: Supplementary file 1 [file biosensors-15-00731-s001.zip › biosensors-3892967-supplementary.pdf]

biosensors

*Supplementary materials*

# An Accumulation Pretreatment-free POCT Bio-chip for Visual and Sensitive ABO/Rh Blood Cell Typing

**Pengcheng Wang [1,†], Mingdi He [2,†], Yan Ma [2], Yunhuang Yang [1,3,*] and Rui Hu [1,3,*]**

1 State Key Laboratory of Magnetic Resonance Spectroscopy and Imaging, Innovation Academy for Precision Measurement Science and Technology, Chinese Academy of Sciences - Wuhan National Laboratory for Optoelectronics, Huazhong University of Science and Technology, Wuhan 430074, China
2 Wuhan Blood Center, Wuhan 430030, China
3 University of Chinese Academy of Sciences, Beijing 100190, China
* Correspondence: yang_yh@apm.ac.cn (Y.Y.); hurui@apm.ac.cn (R.H.)
† These authors contributed equally to this work

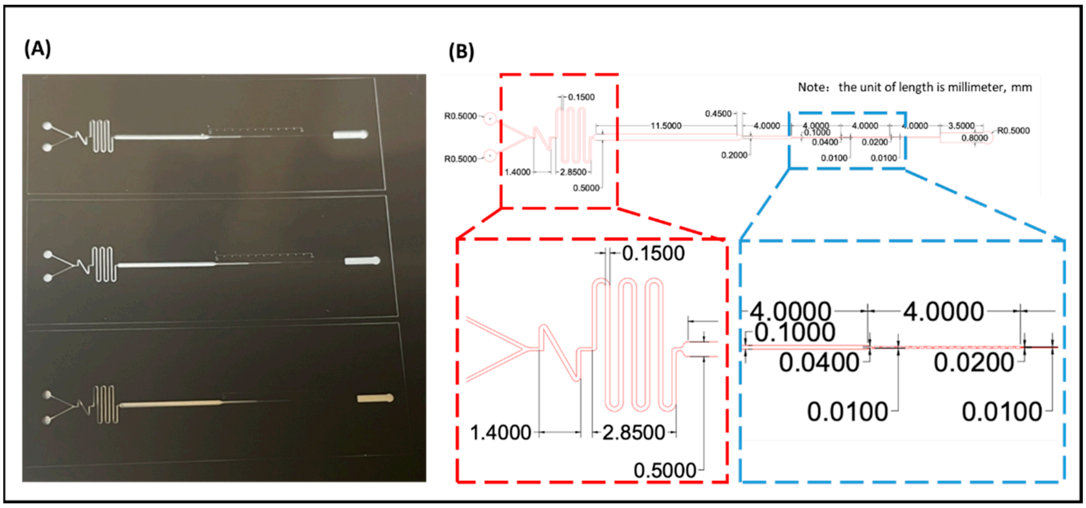


**Figure S1.** (**A**) Image of biochip lithography mask. (**B**) Biochip design dimensioning diagram.

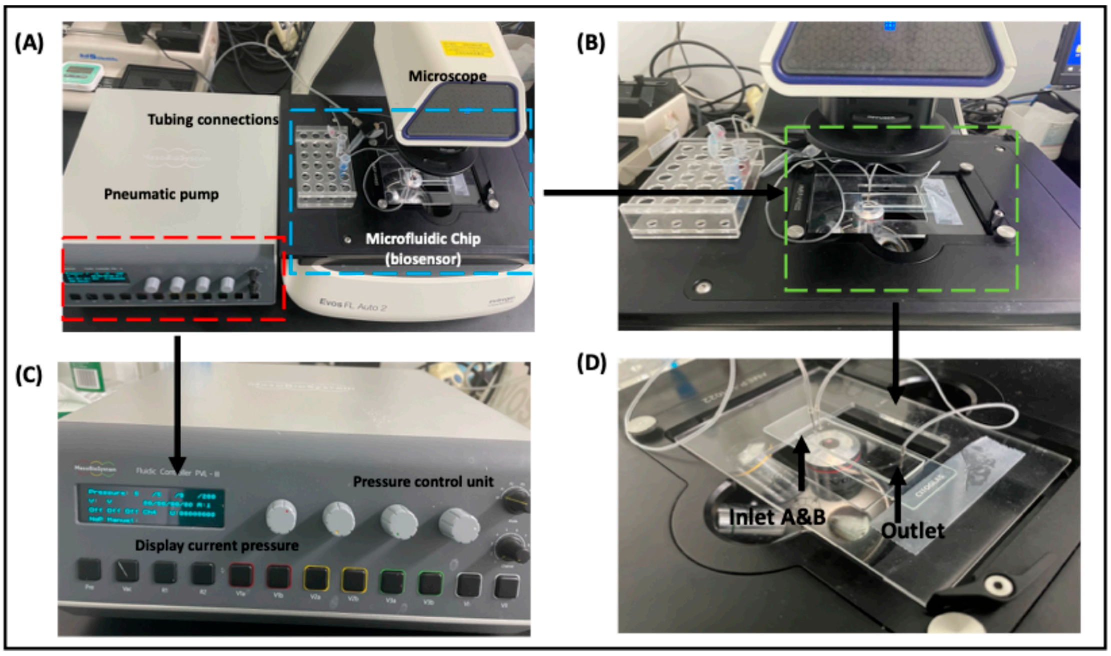


 https://doi.org/10.3390/bios15110731

**Figure S2.** (**A**) Image of testing system of blood type biochip. (**B**) Tubing connection between biochip and pneumatic pump. (**C**) Display panel of pneumatic pump showing the negative pressure. (**D**) The image of biochip under working conditions.

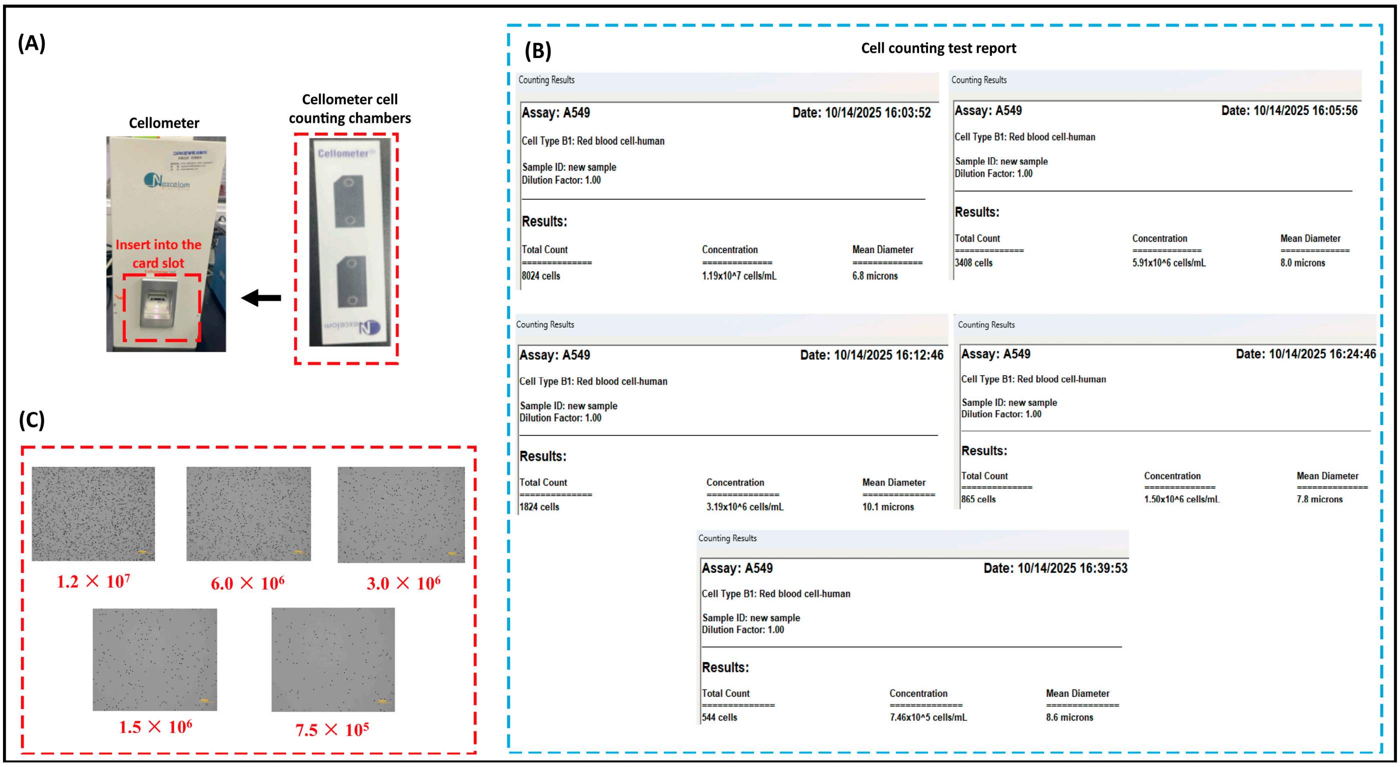


**Figure S3.** (**A**) Image of automatic cell counter and cell counting chamber. (**B**) Testing report of red blood cells (RBCs) obtained using the Cellometer K2 Cytometer. (**C**) Images showing different RBC concentrations.

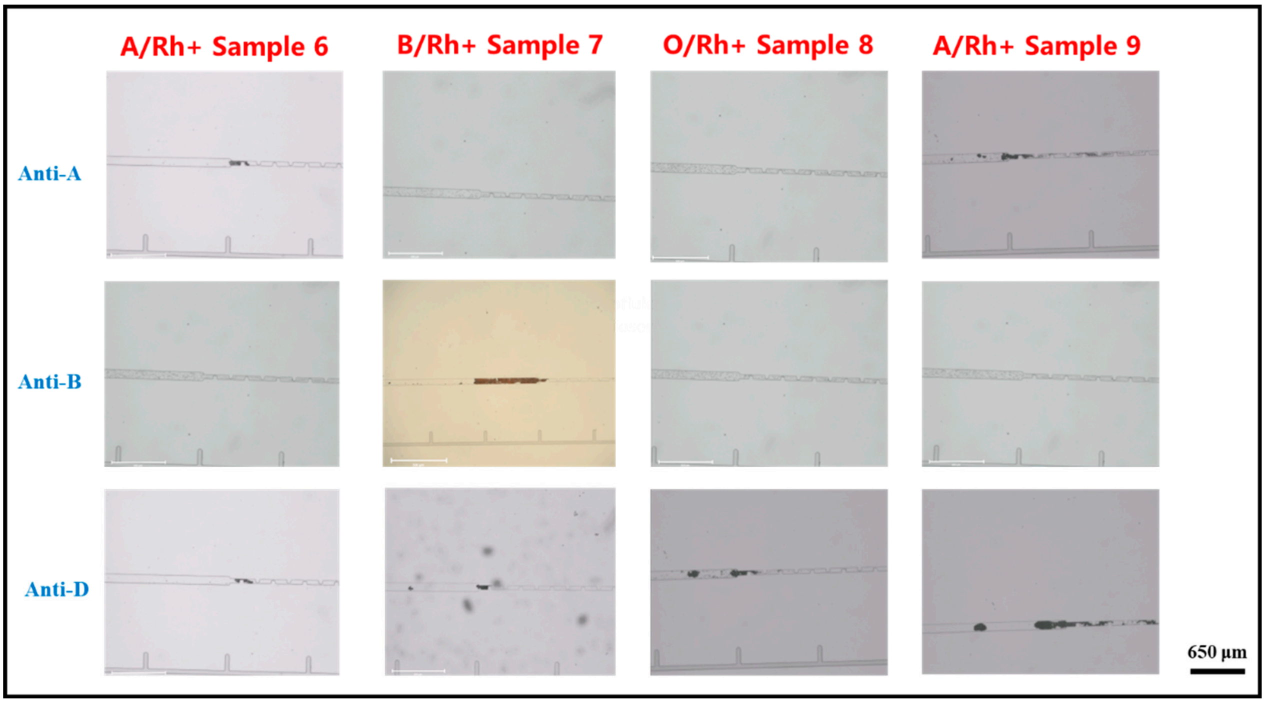


**Figure S4.** Testing results of clinical blood sample for blood typing.

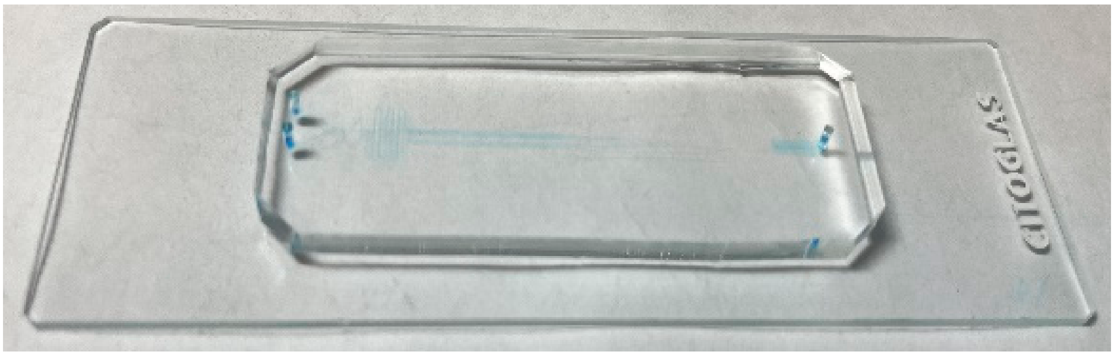


**Figure S5.** Image of biochip filled with colored dye.

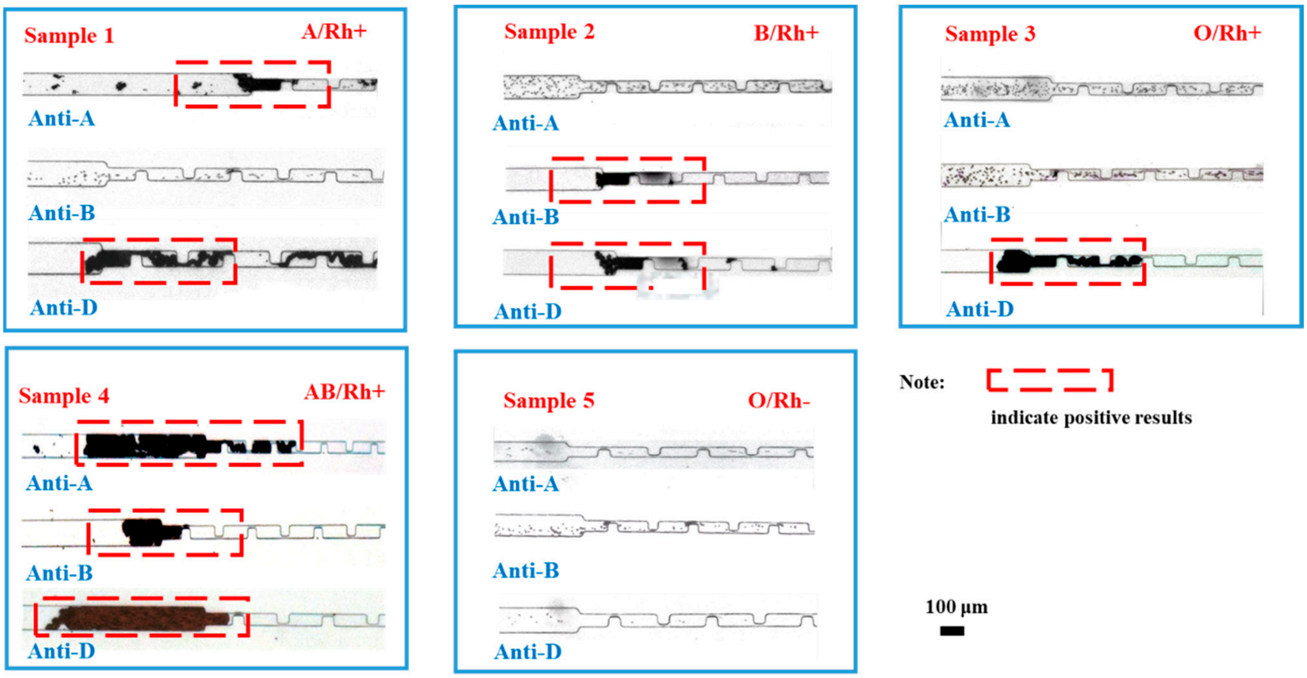


**Figure S6.** Testing results of clinical blood sample for blood typing.

**Table S1.** Comparison of blood typing results obtained using the developed microfluidic biochip and the classical gel column method.

| Sample | Biochip method | | | micro-column gel | | | Result |
|---|---|---|---|---|---|---|---|
| | A | B | D | A | B | D | |
| 1 | + | - | + | + | - | + | A/Rh+ |
| 2 | - | + | + | - | + | + | B/Rh+ |
| 3 | - | - | + | - | - | + | O/Rh+ |
| 4 | + | + | + | + | + | + | AB/Rh+ |
| 5 | - | - | - | - | - | - | O/Rh- |

**Table S2.** Comparison with existing POCT blood typing technologies.

| Blood typing technologies | Cost | Detection time/min | Sample volume | LOD (cell/mL) | Accuracy rate/% | Reference |
|---|---|---|---|---|---|---|
| Paper-based assay | - | 10 | - | - | 85% | [20] |
| LSPR biosensors assay | 0.5$ | 10–20 | 1 mL | $4 \times 10^7$ | 90% | [21] |
| Present method | 0.2$ | 5 | 10 µL | $3 \times 10^6$ | Nearly 100% | |
